# Proton beam irradiation induces invisible modifications under the surface of painted parchment

**DOI:** 10.1038/s41598-021-02993-7

**Published:** 2022-01-07

**Authors:** Katharina Müller, Zita Szikszai, Ákos Csepregi, Róbert Huszánk, Zsófia Kertész, Ina Reiche

**Affiliations:** 1 IPANEMA, Ancient Materials Research Platform, USR 3461 CNRS/MC/UVSQ/MNHN, BP48 Saint-Aubin, 91192 Gif-sur-Yvette, France; 2 Rathgen-Forschungslabor, Staatliche Museen zu Berlin, Stiftung Preußischer Kulturbesitz, Schloßstraße 1a, 14059 Berlin, Germany; 3 Institute for Nuclear Research (ATOMKI), Bem tér 18/c, 4026 Debrecen, Hungary; 4 Ph.D. School in Physics, University of Debrecen, Debrecen, Hungary; 5 PSL University, ENSCP, Institut de Recherche de Chimie Paris – Centre de Recherche et de Restauration des Musées de France, UMR 8247 CNRS/MC, 14 quai François Mitterrand, 75001 Paris, France; 6 New AGLAE, FR 3506 CNRS/MC, C2MRF, 14 quai François Mitterrand, 75001 Paris, France

**Keywords:** Materials science, Physics

## Abstract

Ion beam analysis plays an important role in cultural heritage (CH) studies as it offers a combination of simultaneous and complementary analytical techniques (PIXE/PIGE/RBS) and spatially resolved mapping functions. Despite being considered non-destructive, the potential risk of beam-induced modifications during analysis is increasingly discussed. This work focuses on the impact of proton beams on parchment, present in our CH in form of unique historical manuscripts. Parchment is one of the organic, protein-based CH materials believed to be the most susceptible to radiation-induced changes. Various modification patterns, observed on parchment cross-sections by optical and electron microscopy are reported: discoloration (yellowing), formation of cavities and denaturation of collagen fibers. Considerable modifications were detected up to 100 µm deep into the sample for beam fluences of 4 µC/cm^2^ and higher. The presence of ultramarine paint on the parchment surface appears to increase the harmful effects of proton radiation. Based on our results, a maximum radiation dose of 0.5 µC/cm^2^ can be considered as ‘safe boundary’ for 2.3 MeV PIXE analysis of parchment under the applied conditions.

## Introduction

Art technological studies are motivated by their capacity to contribute to the understanding of the creation process of art works, to identify materials origin and age as well as to better assess the present and future state of preservation in order to develop appropriate conservation measures. Art or archaeological objects are studied by various analytical methods using intense radiation sources like synchrotron^1–4^, neutrons^5^, particle beams^6,7^ or lasers^8–11^. The use of intense beams provides various advantages: high spectral or spatial resolutions, high sensitivity, tunable energy, fast analyses and method coupling^12^.

Since several decades, ion beam analysis (IBA) plays an important role in the analysis of cultural heritage (CH) objects, which is reflected in a steadily increasing number of IBA applications in this field^6,7,13–18^. IBA techniques are based on the interaction of light, high-energy ions (He^2+^ or H^+^) with the atoms of the target material, resulting in three different processes that are exploited in the various IBA techniques. The incident ions can throw out inner-shell electrons of the target atom. The excited atoms then return to their ground state by emitting X-rays, the energies of which are characteristic of the target atoms. This process is used in particle-induced X-ray emission (PIXE) and enables the qualitative and quantitative determination of the materials elemental composition. PIXE is the most used IBA technique in the field of cultural heritage. The impinging ions can also undergo atomic reactions with the target atom leading to the emission of gamma-rays (particle induced gamma-ray emission (PIGE)) or can also be backscattered (Rutherford back scattering (RBS)). Key features for IBA success are its relative simplicity, rapidity and a relatively high sensitivity for lighter elements (with characteristic X-ray energies below 10 keV). IBA facilities have progressively adapted to the requirements of unique and often fragile art and archaeological objects and provide nowadays a routine combination of simultaneous and complementary techniques (PIXE/PIGE/RBS), and mapping capabilities with micrometric spatial resolution^15–18^. They can be performed under atmospheric pressure and are considered non-destructive, as no sampling is required. This is certainly one of the main reasons for their advances in the field of CH studies, which of course require non-invasive analyses. However, the impact of the intense radiation on the material during analysis and the possible risks of material modification are attracting increasing attention of researchers in this field^7,19–23^. So far, the number of published papers on radiation-induced changes in CH studies is very limited in view of the great variety of CH materials and their complexity. Systematic studies of the effects of intense radiation on ancient materials are currently quasi-absent, but are necessary to ensure the choice of non-harmful, safe, measurement conditions for their investigation.

In this paper, the impact of an ion (proton) beam on parchment as representative of CH organic materials is qualitatively studied. Ions lose their energy on their way into a material through electromagnetic interactions with the electrons and the atomic nuclei of the material. Energy transfer from the ion beam to the material can produce several side effects, such as displacing of atoms, heating, chemical changes and changes in microscopic structures, depending on the ion type and energy, flux, fluence and the target material^19,20^. Organic materials are supposed to be most susceptible to beam induced changes, as they are less resistant to electronic excitation or heating. An ionisation due to ion–electron interaction can break chemical bonds and stimulate the formation of new ones and thus change the materials structure^19–21^. The question of the effect of proton beams on various CH materials during IBA, mainly PIXE, was dealt with in several papers, mainly on pigments, but has not yet been investigated exhaustively^7,14,20,22,23^. The aim of the present study is to identify possible proton beam-induced changes in parchment as a function of the radiation dose. The relevant physical quantity for beam-induced modifications is the beam fluence, which is the total number of protons (expressed here as their deposited charge) absorbed by the sample per unit of area and time (C/cm^2^)^7^. The knowledge gained about the impact of proton beams can help establish guidelines for safe PIXE analysis on unique and fragile objects such as historical manuscripts.

**Figure 1:**
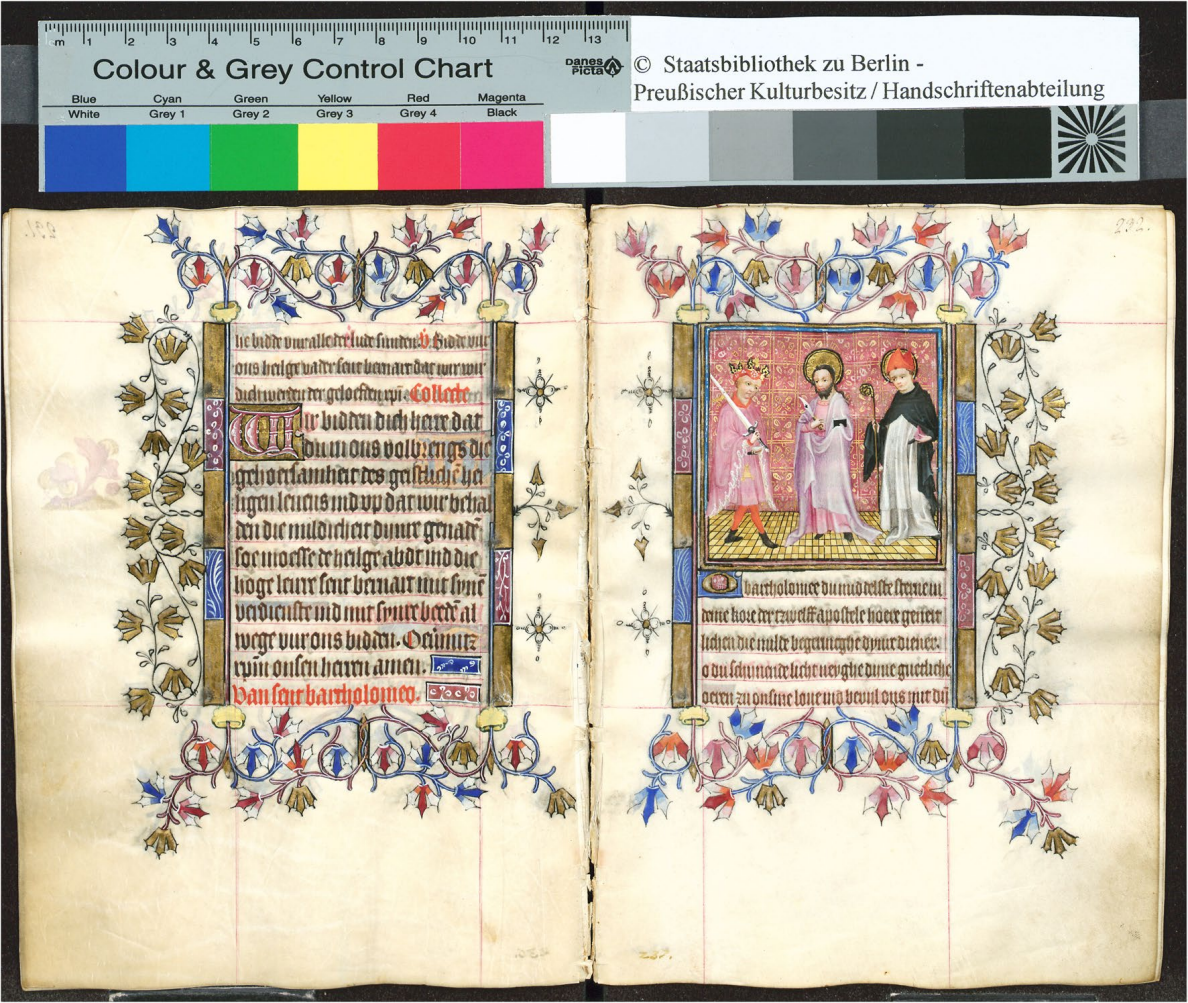
Photograph of a colourful illustrated folio of the Prayer book of Mary of Guelders, 1415 Arnheim (Netherlands), stored in the Staatsbibliothek zu Berlin, Stiftung Preußischer Kulturbesitz (© Staatsbibliothek zu Berlin, M. Hundertmark).

Parchment, made from animal hides, has been used as writing support since Antiquity. Today, we find it in our CH in the form of historical manuscripts, unique written records of our ancestors, some of which are artistically illustrated, the so-called illuminated manuscripts. Most of the surviving manuscripts and books date from the Middle Ages, such as the Prayer book of Mary of Guelders (1415, Netherlands), an exceptional example of illuminated manuscripts with more than hundred colorful miniatures (Fig. 1)^24^. Art-technological studies on illuminated manuscripts were carried out using various spectroscopic techniques such as Raman and infrared spectroscopy, X-ray fluorescence analysis^24–27^ and multispectral imaging^24,28^. Recently, a promising research approach based on a photoacoustic imaging set-up was presented that can be used to reveal hidden content of layered documents^29^. PIXE was used to identify the writing, drawing or painting materials used in old manuscripts^13,14,30–32^. The information obtained by means of PIXE complements art-historical knowledge and contributes to a better knowledge of the creation process and history of acient manuscript. For example, in studies of metalpoint drawings of the Renaissance through the identification of the materials used, insights into artistic techniques and their changes within the creative periods of certain artists could be gained^13,32^. The identification of the pigment and paint palettes used to illustrate the Prayer book of Mary of Guelders made it possible to distinguish between different masters who contributed to its creation^24^. This study was carried out with portable X-ray fluorescence spectroscopy, but in principle it can also be done with PIXE. With the help of PIXE, the composition of iron gall ink, a writing material widely used in old manuscripts, was determined and aging-related changes in the ink and the writing medium were investigated^14,31^. These kinds of study are essential for a better understanding of degradation phenomena and for the development of appropriate conservation measures.

Chemically, parchment is a protein, basically composed of collagen type I. Collagen consists of amino acid sequences (mainly glycine-proline-X or glycine-X-hydroxyproline (X: another amino acid)). Three polypeptide chains are twisted and form triple-helix molecules, which then further arrange together to form fibrils (10–100 nm in diameter), fibres and finally the tissue^26,33^. Parchment degrades over time as evidenced by changes of its physical properties that lead to increased fragility^33,34^. Various factors affect the degree of parchment degradation, as temperature, humidity, mechanical stress, light and/or the presence of microorganisms. There are three major paths for collagen deterioration described in literature: denaturation or gelatinization (unfolding of the triple helix), hydrolysis and oxidation of polypeptide chains which may lead to bond cleavage and breakdown of collagen molecules^33–36^. Damage on historic parchment can be detected at the macroscopic down to the molecular level^36^. The exposure to intense radiation during technical studies, such as proton beams during PIXE analyses, can cause such structural or chemical changes in addition to the “natural degradation”^20,37^.

The parchment samples used in this study were prepared from fresh calfskin as described later on in the materials and methods section. The parchment samples were irradiated with an external proton micro-beam (2.3 MeV, beam size 100 × 100 µm^2^) with beam fluences between 0.5 and 10 µC/cm^2^ corresponding to measurement conditions reported in the literature^20,38,39^, although in some cases even considerably higher deposited charge per area was applied^20,40,41^. The range up to 10 µC/cm^2^ was defined as low beam fluence range and no alterations could be observed below 1 µC/cm^2^ in PIXE studies on paint layers^7^. Since protons transfer most of their energy to the target material at the end of their path, parchment cross-sections were prepared and characterized in order to obtain information about possible changes within the sample that may not be detectable on the sample surface. The preparation of cross-section does not change their micro-morphology. The parchment cross-sections were characterized by means of optical and environmental electron microscopy (OM / ESEM), which are suitable for the visualization of collagen fibers and their possible changes^26,36,42–45^. In addition, Synchrotron-based two-dimensional Fourier Transform Infrared (Sy-2D-FTIR) mapping was used. FT-IR spectroscopy is particularly suitable for characterizing chemical and micro-structural degradation phenomena of parchment-collagen^26,34,35,42,43^ and Sy-2D FTIR mapping on thin sections provides additionally the option to study the distribution of microstructural and chemical changes in collagen with high spatial resolution^46,47^

## Results

**Figure 2:**
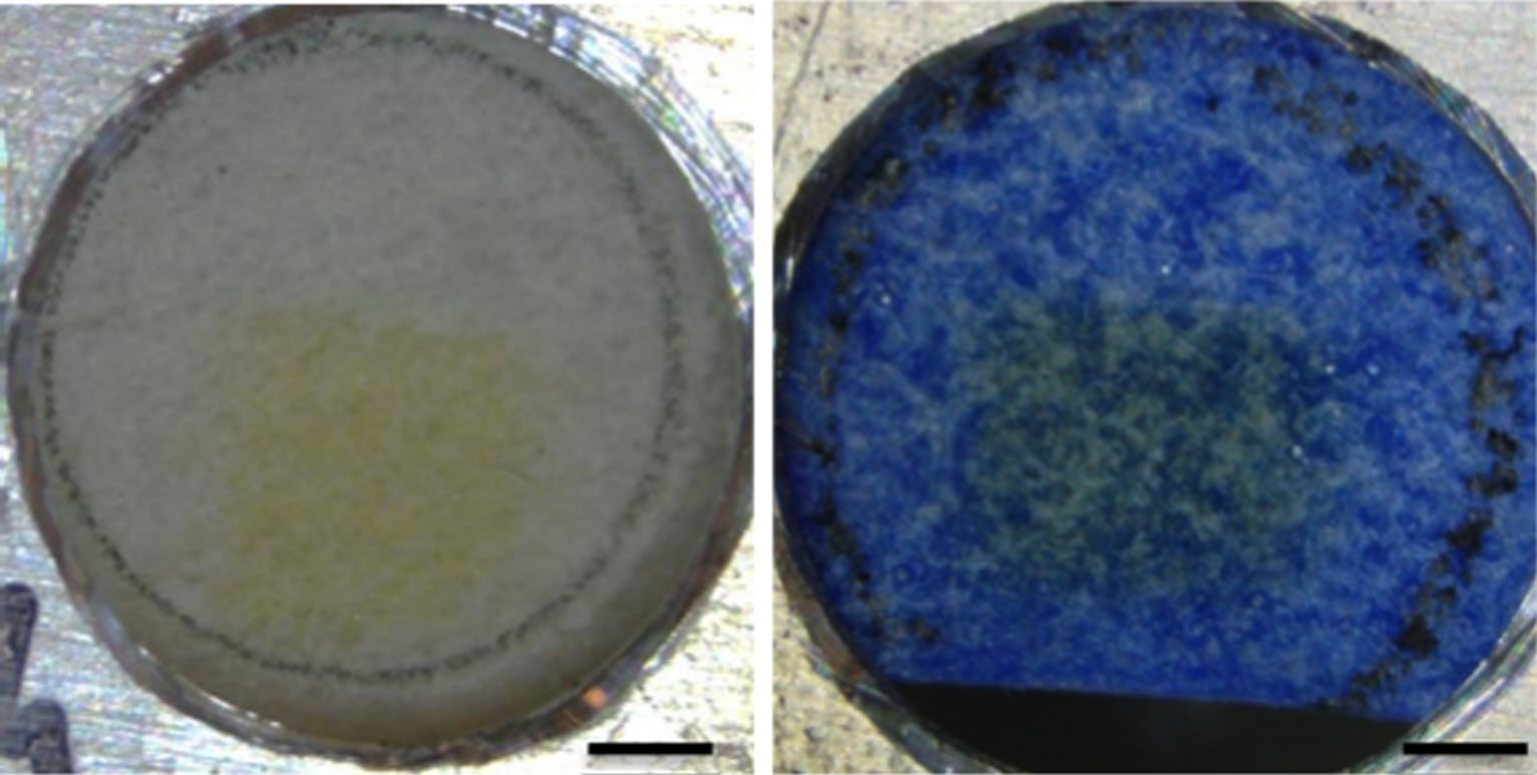
Photographs of parchment samples directly after irradiation with 2.3 MeV energy proton beam. Left: Unpainted parchment, beam fluence 10 µC/cm^2^. Right: parchment with ultramarine paint, beam fluence 4 μC/cm^2^. The circles are 5 mm in diameter. Irradiated spot size is approximately 3.1 × 3.1 mm^2^ (0.1 cm^2^).

Immediately after irradiation, discolorations (yellowing) were visible with the naked eye at the surface of the irradiated areas for all samples (Fig. 2). These stains partly faded out within just a few hours but remained in parchment irradiated with higher beam fluences (4 and 10 µC/cm^2^).

### Micro-morhphological changes observed by optical and electron microscopy

**Figure 3:**
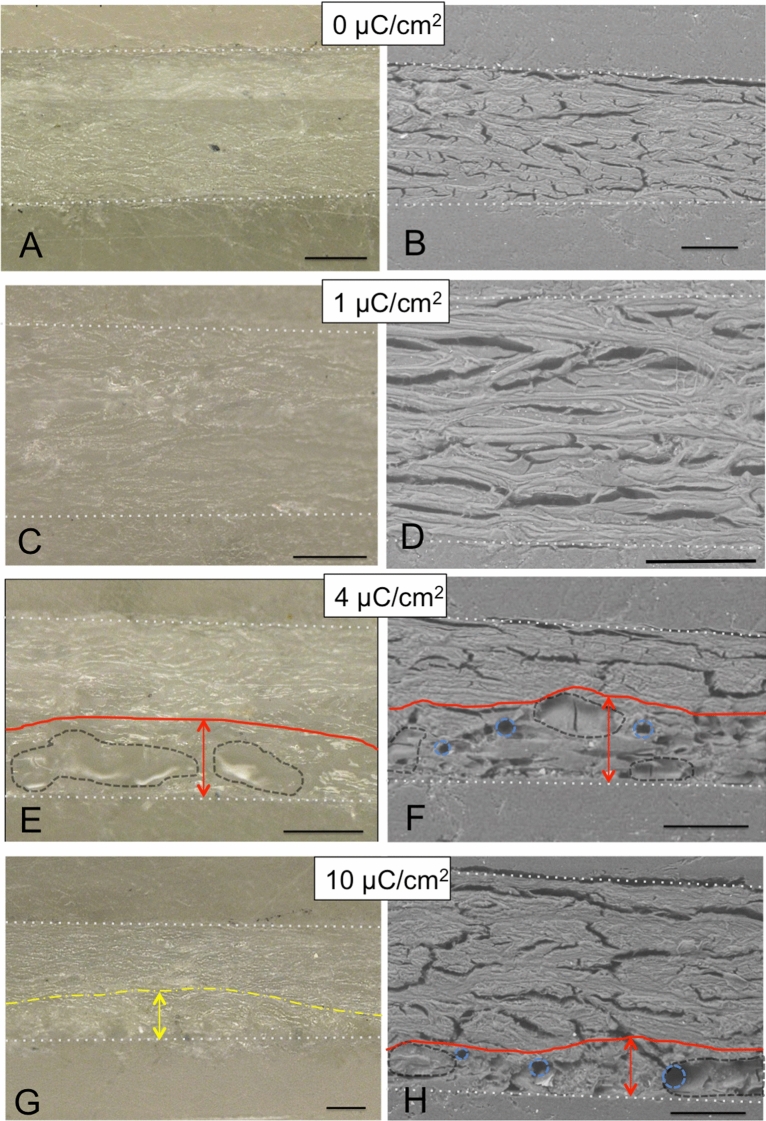
Optical micrographs and back scattered electron (BSE)-ESEM images of parchment cross-sections without paint before irradiation, respectively (**A**,** B**), and after irradiation with proton beam fluences of 1 (**C**,** D**) , 4 (**E**,** F**) and 10 μC/cm^2^ (**G**,** H**). The beam penetrated from the bottom side. The scale bars are 100 µm. The white doted lines indicate the borders of the cross-sections. (**A**) two optical photographs were superimposed to obtain a focused image over the whole sample area. (**E**–**H**) red lines and arrows = zone in which changes were obsereved, dashed line in dark gray = areas with denatured gel-like structure, light blue circles = cavities, yellow dot-dash line = area displaying discoloration. In order to keep the images legible, not all cavities were outlined.

**Figure 4:**
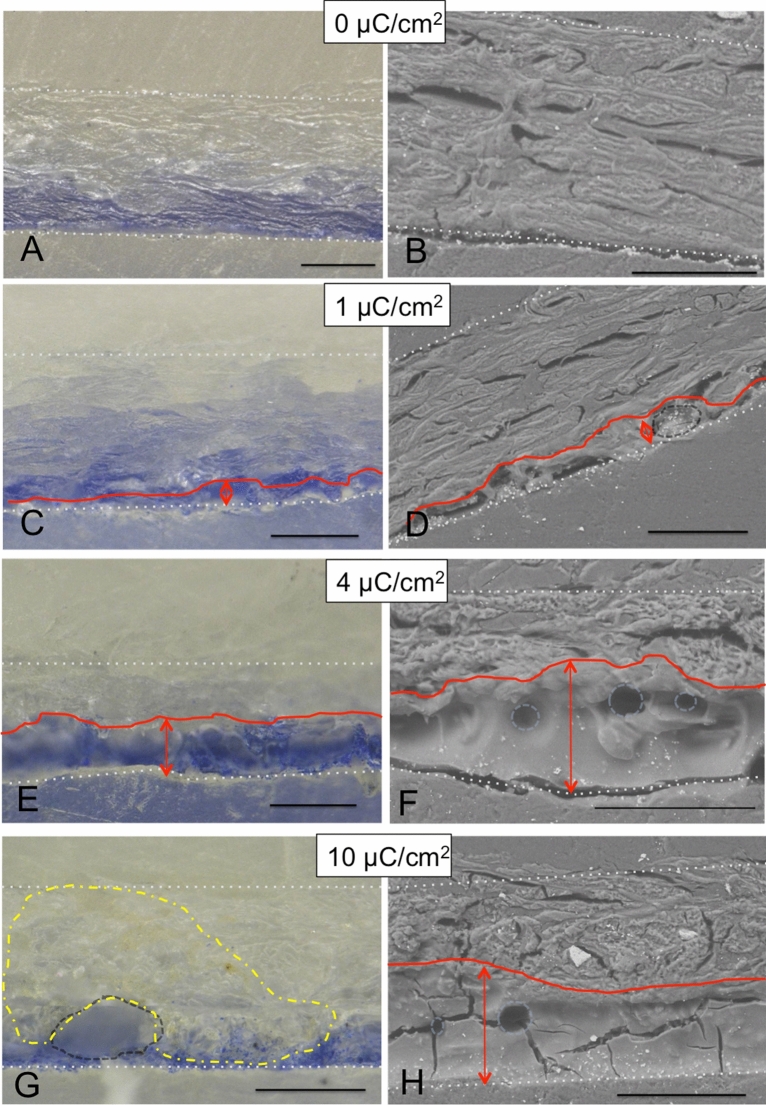
Optical micrographs and BSE-ESEM images of parchment cross-sections with a blue mineral paint layer (ultramarine) before (**A**,** B**), and after proton beam irradiation with estimated charges of 1 (**C**,** D**), 4 (**E**,** F**) and 10 µC/cm^2^ (**G**,** H**). The beam penetrated from the bottom side. The scale bars are 100 µm. The white doted lines indicate the borders of the cross-sections. (**C**–**H**) red lines and arrows = zone in which changes were obsereved, dark gray dashed line = areas with denatured gel-like collagene structure, light blue circles = cavities, yellow dot-dash line = area displaying discoloration. (**F**, **H**) The parchment structure appears amorphous or gel-like in the whole red marked area.

A total of eight parchment cross-sections were examined with OM and ESEM. The obtained images are shown in Fig. 3 for unpainted parchment and in Fig. 4 for parchment with a layer of ultramarine paint. In the figures, the observations for non-irradiated samples and samples that have been irradiated with different radiation doses are compared with one another. In all figures, the proton beam hit the sample from below and the red lines and red arrows indicate the areas in which changes were observed. Table 1 summarizes the OM and ESEM observations for all samples examined.

**Table 1:** Summary of morphological and structural changes observed by OM/ESEM for unpainted parchment sample and parchment with ultramarine paint layer.

Sample (thickness)	Fluence µC/cm^2^	Observed changes	Depth of observation
Unpainted parchment (250 µm)	1	No changes observed	
	4	Collagen fibre structure partly lost (partly gel-like structure)	0–100 µm
		Formation of cavities (10–20 µm in diameter)	0–100 µm
	10	Collagen fibre structure disordered (broken fibers near the surface)	0 – 50 µm
		Formation of cavities (10–30 µm in diameter)	0–50 µm
		Yellowish discoloration	0–ca. 100 µm
Parchment with ultramarine paint (160 µm)	1	Uneven parchment surface	
		Collagen fibre structure disordered	0–10 µm
		Slight shift of pigment grains?	0–10 µm
	4	Complete lost of collagen fiber structure (gel-like structure)	0–100 µm
		Formation of cavities (10–20 µm in diameter)	50–70 µm
		Migration of pigment grains	0–70 µm
	10	Complete lost of collagen fiber structure (gel-like structure)	0–100 µm
		Formation of cavities (10–20 µm in diameter)	50–70 µm
		Migration of pigment grains	0–70 µm
		Yellowish discoloration	0–160 µm (entire cross-section)

For the unpainted parchment significant changes were observed for beam fluences of 4 µC/cm^2^ and higher. In an area between the irradiated surface to a depth up to 100 µm in the sample, the collagen fiber structure seemed to be partially changed (see dark gray bordered areas in Fig. 3E,F,H) and numerous small cavities have formed (see light blue circles in Fig. 3F,H). For the highest beam fluence (10 µC/cm^2^), a yellowish discoloration was found in the interior of the parchment (see yellow marked area (line and arrow) in Fig. 3G).

**Figure 5:**
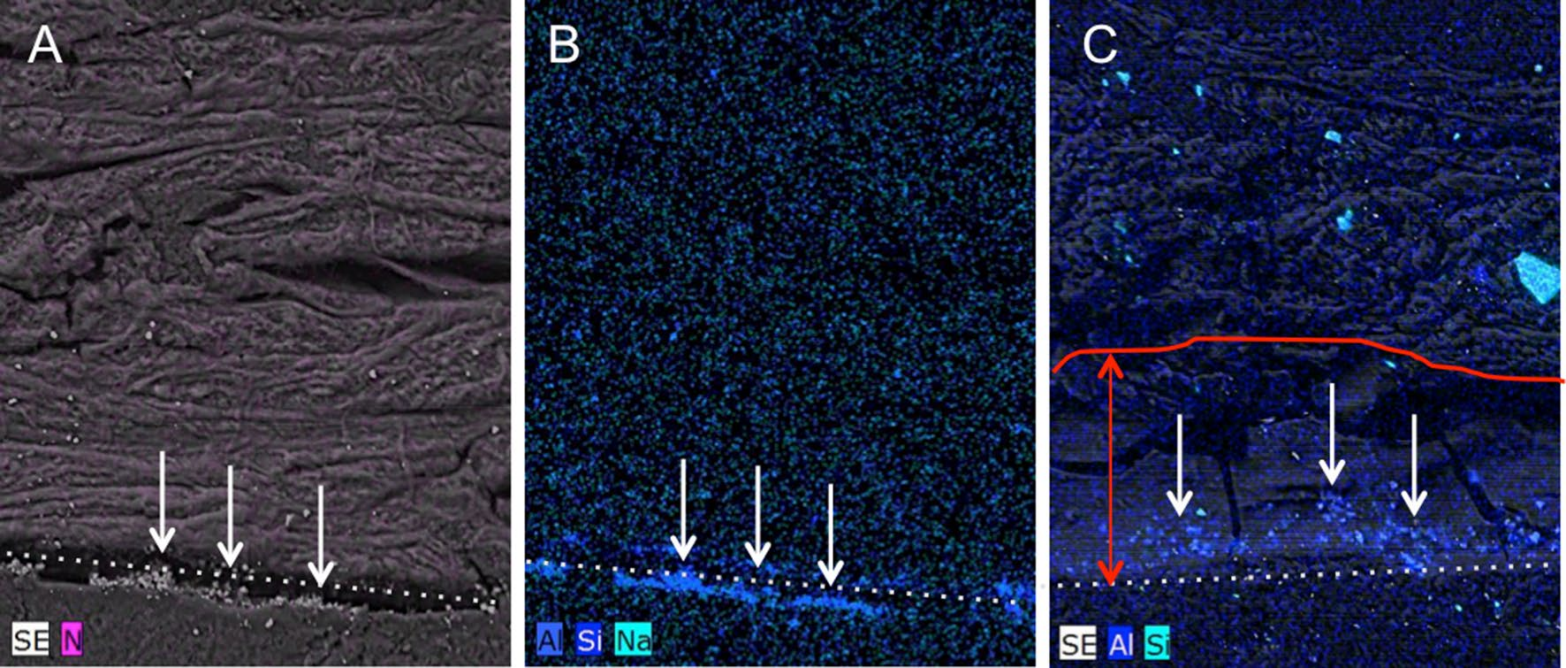
Chemical distribution maps for Si and Al obtained by chemical microanalysis (ESEM-EDX) for parchment samples with ultramarine paint layer: (**A**, **B**) not irradiated and (**C**) irradiated with a charge of 10 μC/cm^2^. For better interpretation SE images were added reflecting the surface morphology of the sample. The white arrows indicate the location of the Al- and Si-rich grains. Automatic color assignment causes the elements to have different colors in the images. (**C**) The Na concentration was obviously near or below the LLOD and was therefore not considered for the irradiated sample. The red line and arrow mark the zone in which changes were observed, like in Fig. 4h.

Considerably more drastic changes were observed for the parchment samples with ultramarine paint layer. In contrast to the unpainted sample, slight changes of the parchment morphology occured at the parchment surface even at a fluence of 1 µC/cm^2^ (Fig. 4C,D). At higher beam fluences (4 and 10 µC/cm^2^) the parchment has apparently completely lost its typical collagen fiber structure up to a depth of 100 µm (see areas outlined in red in Fig. 4F,H). Within this zone, the parchment has a gel-like structure (amorphous zone looking like melted). Cavities were formed, correspondingly to the energy deposition pattern of protons, not on the parchment surface but about 50 to 100 µm below (see light blue circles in Fig. 4F,H). A yellowish discoloration of the parchment material extended over the entire cross section of the sample, which was irradiated at 10 µC/cm^2^ (see yellow marked area in Fig. 4G). However, it seems that the colour of the pigment itself did not change due to the irradiation. In addition, the ESEM images showed that ultramarine pigment grains seem to have shifted within the amorphous zone into the interior of the parchment. Figure 5 shows chemical distribution maps for Si and Al obtained by elemental microanalysis using the energy dispersive X-ray analyser (EDX) installed in the ESEM. These two elements are characteristic of the composition of ultramarine, more precisely of lazurite with the chemical formula (Na, Ca)_8_(AlSiO_4_)_6_(SO_4_, OH, S, Cl)_2_^48^, which is a main component of ultramarine, responsible for its colour. It can be seen from the distribution images that the pigment grains in the non-irradiated reference sample are on the surface of the parchment (Fig. 5A,B), while in the irradiated sample they appear to have migrated into the parchment (Fig. 5C). It is likely, that the displacement of the grains is due to changes in the collagen structure induced by proton irradiation and not to the sample processing because all samples have undergone the same preparation procedure and this phenomenon was not observed for the not irradiated parchment.

### Micro-structural or chemical degradation of parchment assessed by Synchrotron-based-two-dimensional Fourier Transform Infrared mapping

Synchrotron-based 2D-FTIR mapping was applied to a parchment irradiated with only 0.5 μC/cm^2^ (W7-A2) as well as a parchment reference sample to verify if there were chemical or micro-structural changes that cannot be detected by OM/ESEM observations.

Relative positions and intensities of specific vibration bands of collagen, the main constituent of the parchment, found in IR spectra are indicative of its state of preservation: these are above all the amide I and amide II bands at about 1650 cm^−1^ (C=O stretching vibration) and 1550 cm^−1^ (mainly N–H bending vibration), respectively. First, an increase of the intensity ratio of amide I to amide II bands indicates hydrolysis of polypeptide chains. Second, a characteristic increase of the frequency difference between the amide I and amide II bands due to a shift of amide II band to lower frequencies, is a sign for the denaturation of collagen structure^33,35,42,43^. Third, the appearance of a vibration peak between 1750 and 1700 cm^−1^ may indicate the oxidation of polypeptide chains. The evaluation and interpretation of FTIR spectra with regard on the degradation of collagen are discussed in more detail in the Supplementary (see Supplementary Information S.2, Supplementary Table S2 online).

**Figure 6:**
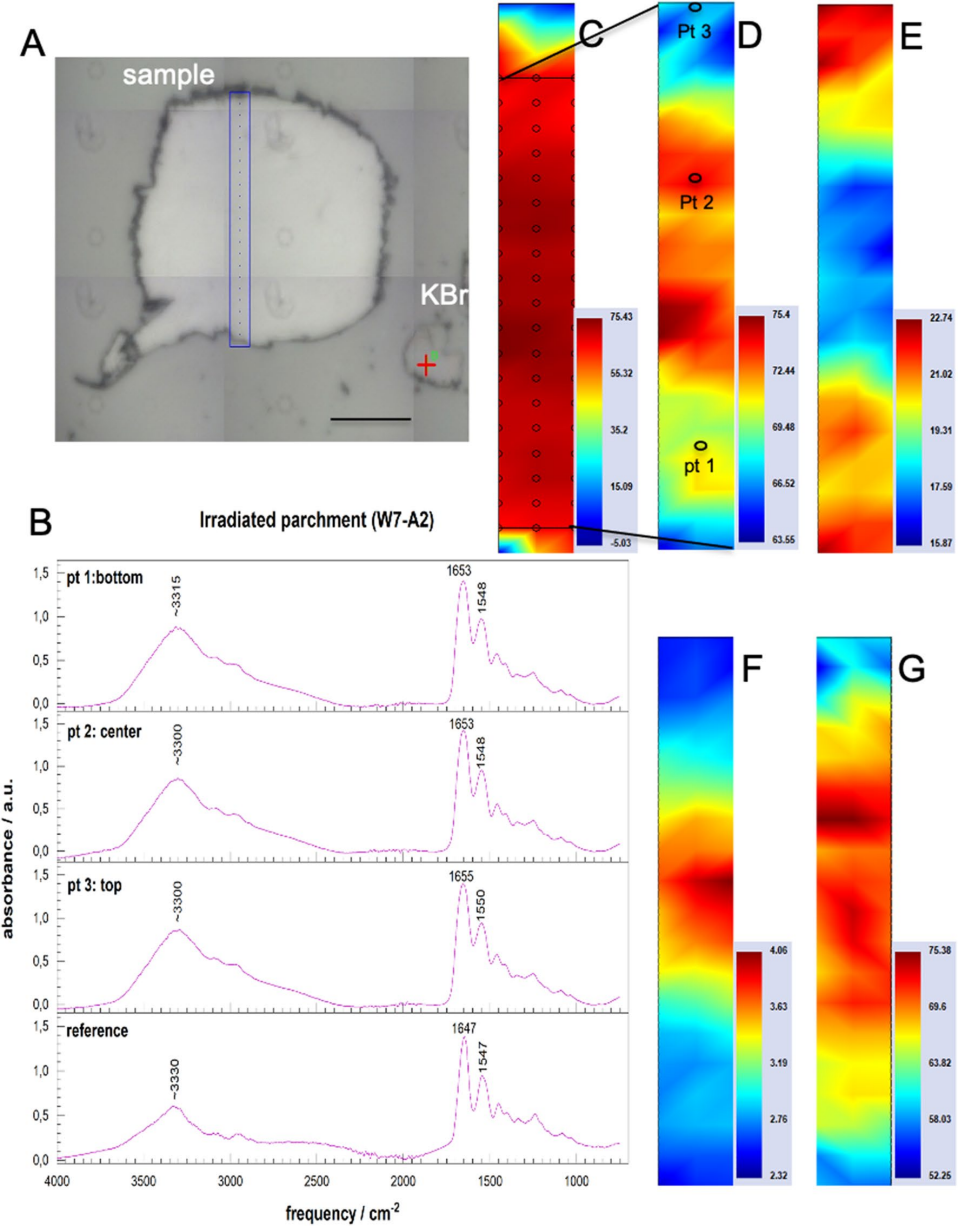
Results of Sy-FTIR 2D mapping on irradiated parchment sample (W7-A2, 0.5 µC/cm^2^). (**A**) Optical micrograph of the thin sections placed in a diamond pressure cell with the scanned area indicated (blue rectangle). The scan parameters were: 3 × 24 measure points, aperture 12 × 12 μm^2^, step size 12 μm, 64 scans accumulated. KBr grains were added to measure the background. (**B**) Selected FT-IR spectra with indication of representative vibration bands: amide I at about 1650 cm^−1^, amide II band at about 1550 cm^−1^ and the water band at about 3300 cm^−1^. (**C**–**G**) 2D chemical maps representing the intensity distributions of: (**C**) Amide I band. The black rectangle indicates the ROI used for semi-quantitative evaluation. (**D**) Amide I band within the ROI zone. (**C**) Amide II within the ROI zone. (**E**) Intensity ratio of the amide I to amide II bands within the ROI zone. (**F**) Water band within the ROI zone.

Figure 6 illustrates the results of Sy-2D-FTIR mapping obtained for the irradiated parchment sample (W7-A2, 0.5 µC/cm^2^). Details on the Sy-2D-FTIR mapping results for the not irradiated parchment reference are given in the Supplementary (see Supplementary information S.1, Supplementary Fig. S1 online). Table 2 summarizes the results of the semi-quantitative evaluation of the 2D-FTIR maps for both the irradiated parchment sample and the reference sample.

**Table 2:** Semi-quantitative evaluation of the Sy-2D-FTIR data for the parchment sample irradiated with 0.5 µC/cm^2^ (W7-A2) in comparison to the not irradiated reference sample.

Sample		ν amide I (cm^−1^)	ν amide II (cm^−1^)	Δ (ν amide I–ν amide II) (cm^−1^)	A amide I/A amide II	A OH /NH/A amide I
Reference	Min	1641	1543	94	2.2	0.8
	Max	1662	1559	112	4.1	1.3
	Mean ± stdv RSD (%)	1655 ± 4	1550 ± 3	105 ± 44	2.9 ± 0.620	1.0 ± 0.113
W7-A2	Min	1649	1542	102	2.4	0.83
	Max	1658	1550	112	4.1	1.03
	Mean ± stdv RSD (%)	1654 ± 2	1548 ± 2	106 ± 32	3.1 ± 0.414	0.95 ± 0.045

The microscope image in Fig. 6A illustrates the scanned area on the thin section of this sample. Figure 6B shows IR spectra of the reference sample and of three selected analysis points of the irradiated sample, each of which is representative of different areas in the 2D distribution images (see Fig. 6D). The FTIR spectra appear to be almost identical, with the exception of slight shifts in the band positions of the amide I band to higher frequencies for the irradiated sample. They do not show any vibration bands between 1750 and 1700 cm^−1^ (C=O stretching vibrations). The formation of additional carbonyl groups through oxidation of polypeptide chains can thus be excluded.

The obtained 2D chemical maps of the characteristic collagen bands are presented in Fig. 6C–G. They reveal certain heterogeneities for the amide I and amide II bands within the ROI chosen for semi-quantitative calculations (Fig. 6D–G). These fluctuations in peak intensities lead to variations in the band area ratios between about 2 in the inner part and 4 in the outer part of the cross section. However, these values are in the range of the values found for the parchment reference sample (Table 2).

The band positions show slight variations for both amide peaks within the scanned area (Table 2). However, these variations are within the range of fluctuation of the band positions of the reference sample. The peak positions averaged over the ROI area do not differ between the irradiated and non-irradiated parchment samples (Table 2). Consequently, no characteristic increase of the frequency difference (ν amide I–ν amide II) was observed for the irradiated sample, that could indicate a denaturation of collagen structure due to the beam.

In addition, Fig. 6G shows the chemical 2D FTIR map for the water vibration band in the frequency range around 3300 cm^−1^. In the outer areas the values are lower as in the inner part of the parchment cross-section. This may indicate a decrease of water content in these areas. External dehydration could be due to unsuitable storage and transport conditions.

## Discussion

### Discoloration

Discoloration, mainly of pigments, has been discussed extensively in literature and generally attributed to the formation of color centers^7,20,22,23^. In parchment, the formation of color centers is enabled by remnants of substances that were used for parchment processing, like lime or chalk^23^. However, the formation of color centers probably only played a subordinate role in our case. The discolorations were mainly caused by the chemical action of the ion beam on the organic material. During the excitation and ionization by the proton beam, organic radicals, double bonds and other conjugated bond systems are formed, whereby a shift in the UV absorption range becomes visible^49,50^. The material can relatively easily return to its original state through recombination or reaction with oxygen, whereby the yellowing disappears again. Yellowing was observed immediatly after irradiation at the parchment surface but also a few weeks later inside the material, with the colour of the pigment itself appearing unchanged. This is in line with a literature study in which the authors observed the formation of stains during PIXE analysis of illuminated manuscripts, but never in the analyzed pigment itself, but on the back of the parchment^23^. This can be explained by the fact that the energy loss of the protons at the end of their pathway through matter is maximal, below the thin layer of pigment. The estimated penetration depth of protons with 2.3 MeV is 100 µm based on SRIM calculations for various modell matrices, as pure collagen, collagen with different water contents and skin^51^.

### Denaturation of collagen structure and formation of cavities

Protons lose their energy on their way through matter as a result of interactions with the electrons (ionization) and atomic nuclei (collision) of the material^19,20^. In parchment, these processes can break chemical bonds and stimulate the formation of new ones. Breaking of cross-links and the release of hydrogen bonds, responsible for the stability of the triple helix, can lead to considerable disruptions in the hierarchical structure of collagen molecules and fibers. The ordered collagen structure can collaps and transform into a random coil and finally a gel-like structure^33,44,52^. These degradation processes possibly took place in the parchment during irradiation with proton beam fluences of 4 µC/cm^2^ and higher. They caused the collagen fiber structure to transform into gel-like structure in places or in the entire penetration area of the beam, as was especially observed for the parchment with the ultramarine paint layer. In this study, no signs of collagen degradation due to the beam were observed after irradiating the parchment with a 2.3 MeV proton beam with beam fluence of only 0.5 µC/cm^2^.

The formation of cavities could be due to the development of gas bubbles, which is a very typical effect for intense H- and He-beams^20^. Breaking and reorganising of chemical bonds results also in the release of small gaseous molecules, such as hydrogen molecules or carbon oxides, wich can diffuse and leave the material^19^. This effect may be at its maximum at the end of the proton pathway, which could explain the fact that cavities were observed particularly inside the matter and less near the sample surface.

Both, the denaturation of the hierarchical collagen structure into a gel-like amorphous structure and the formation of bubbles could enable or even cause the movement of the pigment grains within the degraded zone of the parchment tissue.

The heating effect of the irradiation can additionally be very critical for organic materials as they are often very poor thermal conductors^19,20^. A study on the assessement of surface heating during ion beam irradiation revealed a rise of surface temperature up to approx. 110 °C for hair, a protein-based biological material, with a 2.5 MeV/20 nA proton beam, applied in vacuum chamber^53^. Accelerated aging experiments to assess hydrothermal stability of parchment revealed that oxidation caused by heat (150 °C) in the absence of water resulted in severe damage at microscopic level up to a full degradation of the fiber mass, transforming it into gel-like fragments^52^. Another study on the thermal denaturation of collagen in a dry environment reported a two-step transition (at 150 °C and 220 °C) that ultimately led to conformational changes in the collagen molecules from the triple helix to the random coils^52^. The minimization of the heating effect is one reason why measurements in air are preferred for sensitive materials, although this leads to a certain loss of analytical performance with regard to the PIXE detection limits and the beam resolution. Of course, the energy deposition pattern of ions (predominantly below the surface) further complicates the situation.

### Effect of paint layers

Why are radiation-induced changes in parchment more pronounced when the parchment surface is covered with ultramarine paint? This is an interesting research question, beyond the scope of this article, but will be explored in further research*.* However, we believe that the main reason for the increased denaturation of collagen for the parchment painted with ultramarine is probably related to the fact that the layer of pigment seals the surface like a kind of varnish. This would mean that volatile compounds that form during irradiation cannot be easily evacuated and additional thermal insulation is created. However, this hypothesis is so far based on only a limited number of samples and requires further studies, possibly including different pigment types, to confirm the first observations.

## Conclusions

The in-detail examination by OM and ESEM of irradiated parchment samples highlighted considerable modifications for proton beam fluences of 4 and 10 µC/cm^2^, but also slight changes for lower collected charges (1 µC/cm^2^) when the parchment has been covered with an ultramarine paint layer.

The modification patterns for parchment painted with ultramarine paint is much more pronounced as the one observed for not painted parchment. This study has clearly shown that a layer of a mineral paint has an influence on the modification potential of the proton beam on the organic carrier underneath. The underlying processes must be examined more closely in ongoing studies.

Some radiation-induced change features may not be visible at the sample surface, as the formation of cavities and the denaturation of collagen fibers, which were observed up to 100 µm deep into the sample. This could especially be the case when the parchment surface has been painted with pigment-based paints. In order to be able to assess whether the material was modified by the radiation, it is obviously not enough to look at the material surface, because changes can occur invisibly inside the sample.

Based on our results, an irradiations dose of 0.5 µC/cm^2^ does not cause any micro-structural or chemical changes in unpainted parchment under the here applied conditions. Consequently, this dose could be defined as a first proxy of a ‘safe boundary’ for PIXE analyses of illuminated parchment manuscripts. But, the presence of pigment-based paints on the parchment surface seems to influence the material-changing effects of proton radiation. This aspect should be taken into account when determining safe analytical conditions.

Even if the reported effects of the proton beam on parchment based only on observations of a limited number of samples, they nevertheless show the possibility of damage of parchment under the analytical conditions used and urge caution. This study is a basis for ongoing research in the frame of the IPERION HS European project. We are planning to pursue this research in order to improve the knowledge of beam-induced changes and the underlying processes.

Probably, change can likely never absolutely be eliminated but minimized by optimizing experimental parameters to obtain maximum ratio of gained information to possible material modification. Further investigations of radiation-induced change of parchment samples using optical coherence tomography (OCT) will be discussed in an upcoming paper (Csepregi et al. forthcoming). Another well adapted method would be investigation with multiphoton microscopy^54^.

## Materials and methods

### Preparation of parchment samples

The samples in this study originate from fresh parchment prepared from calfskin. Prior to the application of the paint layers the parchment was treated as follows in order to thin it down and to smoothen its surface: scraping the surface with a glass shard, polishing with abrasive paper (grit size 600) and squeezing with an agate stone. The parchment had a relatively heterogeneous thickness of 150 to 370 µm. The blue paint was produced by thoroughly grinding the ultramarine blue pigment (*Kremer Pigments*) in a mortar and then mixing it with a suitable proportion of binder, a solution of 2 g of gum arabic powder (*Kremer Pigments*) in 5 ml of deionized water. The weight ratio was about 6 parts of pigment to 100 parts of binder. Ultramarine was chosen for this study because it was identified as blue pigment in the decorative frame of the medieval manuscript "Prayer Book of Mary of Guelders"^24^.

After proton beam irradiation the parchment samples were embedded in epoxy resin (Epofix, Stuers), cut to obtain cross sections that then were polished with abrasive paper (granulations up to 3200). For Sy-2D-FTIR mapping the embedded cross-sections were cut by means of a microtome to obtain thin sections of 6 µm thickness.

### Irradiation of parchment samples

**Figure 7:**
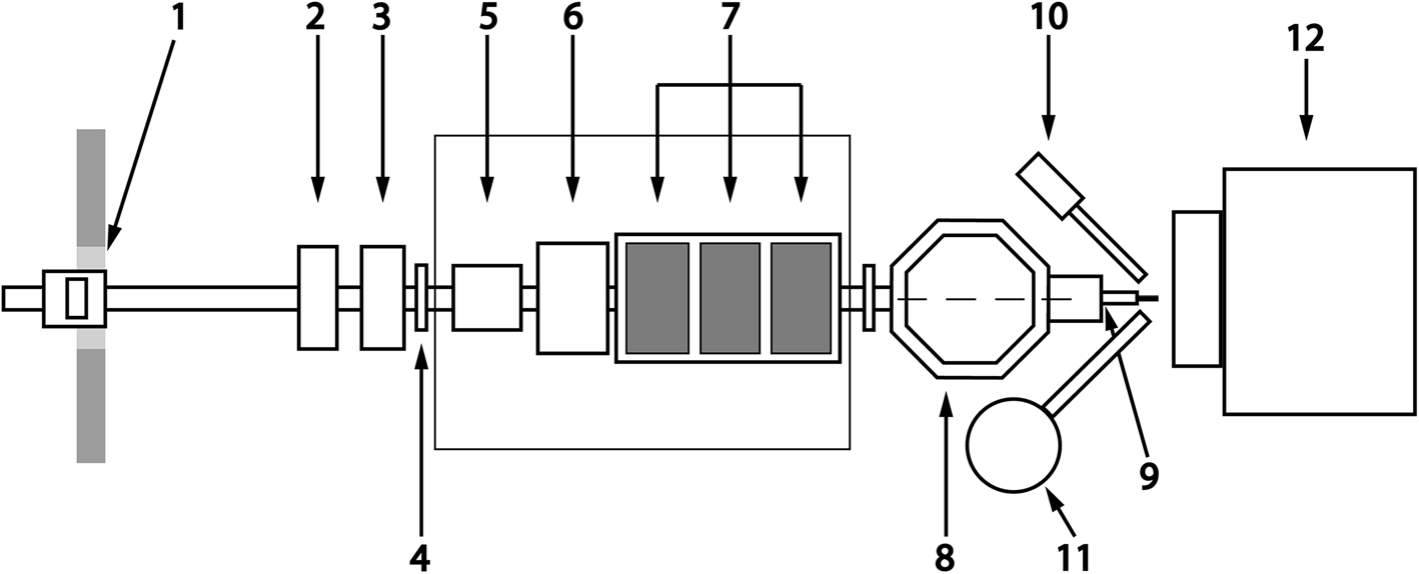
Schematic representation of the micro-PIXE set-up in ATOMKI. (1) Object slits, (2) pneumatic valve, (3) quartz viewer, (4) fast valve, (5) collimator slits, (6) scanning coils, (7) quadrupole triplet, (8) vacuum chamber, (9) exit nozzle with 2 lasers, (10) SDD X-ray detector, (11) Si(Li) X-ray detector, (12) precision XYZ-stage.

The parchment samples were irradiated with a focused proton beam extracted in the air in the ATOMKI Scanning Nuclear Microprobe facility using the in-air micro-PIXE setup^55^. Figure 7 shows a schematic description of this set-up. The energy of the irradiating protons was 2.3 MeV on the surface of the sample. The beam with a size of 100 × 100 μm^2^ was scanned over an area of 3.1 × 3.1 mm^2^. The beam current was 0.5 nA. The radiation dose during the measurement was monitored with a beam chopper. For beam-induced modifications, the relevant physical quantity is the fluence, which is the total number of protons per unit area that has impacted the sample expressed in µC/cm^2^^7^. In this study beam fluences of 0.5, 1, 4 and 10 μC/cm^2^ were applied. For each radiation dose, only one sample was available for the analyses, each with an irradiated area of approx. 1 cm^2^ in diameter. The number of samples was limited due to the restricted access to beam time.

Table 3 provides information on the studied parchment samples, the respective radiation doses and the analytical techniques used to examine the samples.

**Table 3:** List of parchment samples with details of the respective radiation doses and analysis techniques used.

N° Sample	Paint layer	Beam fluence μC/cm^2^	Applied analytical techniques
Reference	None	0	SR-FTIR mapping
W8–B5	None	0	OM, ESEM
W7–A2	None	0.5	SR-FTIR mapping
W7–A3	None	1	OM, ESEM
W8–B2	None	4	OM, ESEM
W8–B4	None	10	OM, ESEM
Rb05	Ultramarine/gum Arabic	0	OM, ESEM, ESEM-EDX
B4–C4	Ultramarine/gum Arabic	1	OM, ESEM
B5–D3	Ultramarine/gum Arabic	4	OM, ESEM
B6–E4	Ultramarine/gum Arabic	10	OM, ESEM, ESEM-EDX

### Optical and electron microscopy

The assessment of parchment degradation features by optical and electron microscopic methods provide information on the collagen fibre morphology and structure. For optical microscopy (OM) the digital microscope Keyence VHX-500FD was used to take microscopic images with 100 × to 700 × magnification.

The environmental scanning electron microscope (ESEM) Quanta 200 from FEI, was applied for the investigation of parchment samples in the low vacuum mode, not requiring conductive coating of the sample. The measurement conditions were as follows: accelerator voltage 15 kV, spot size 6.0 or 7.0 nm, emission current 100 μA and the vacuum 0.53–0.82 Torr. For each cross-section, between 17 and 31 back scattered electron (BSE) images were taken at different magnifications (from 50 × to 2200 ×) and at different points on the sample in order to be able to document the entire sample area. The microscope was equipped with an energy dispersive X-ray analyser XFlash 4010 from Bruker AXS Inc. enabling qualitative elemental microanalysis and imaging (ESEM-EDX). Using EDX, it is possible to gain quickly information on the chemical composition of the sample and the distribution of the elements that are present. EDX analyses were carried out at three different points for three parchment samples with a blue paint layer in order to deduce the composition of the light grains, visible on the BSE images, as ultramarine pigment grains (lazurite) from their characteristric components Al and Si.

### Fourier-transform infrared spectroscopy (FTIR) and 2D mapping (Sy-2D-FTIR mapping)

High spatial resolution synchrotron 2D FTIR mapping was conducted at the IRIS beamline at BESSY II/ HZB in Berlin-Adlershof. A Nicolet 8700 spectrometer was used coupled with a Nicolet Continuμm Infrared microscope from ThermoFisher Scientific, equipped with a 32 × objective. For Sy-2D-FTIR mapping thin sections (6 μm thin) were cut from the embedded cross-sections using a microtome at the laboratory of IRIS beamline, BESSY II/HZB. The thin sections were pressed in a diamond cell (High Pressure Diamond Optics) for measurements. Spectra were acquired in transmission mode using Omnic software under the following conditions: aperture (12 × 12) or (15 × 15) μm^2^, step size 12 μm, 64 scans accumulated for each spectrum, spatial resolution of 4 cm^−1^ and a spectral range of 600–4000 cm^−1^. Since the beam time available for the examination of the irradiated parchment samples was very short, only two samples could be analyzed with the help of Sy-2D-FTIR mapping. Two maps were conducted per sample. Each Sy-2D-FTIR map is composed of about 60 single measurements.

The obtained Sy-2D-FTIR mapping data were evaluated using *CytoSpec* software. Prior to calculating band intensities, the spectra were subjected to the following pre-treatment procedure: cutting off the frequency range below 750 cm^−1^, baseline correction using polynomial fit 2nd order, smoothing (*Savitzky-Golay*, 11 smoothing points). The intensities of the amide I, amide II and amide A (or water) bands were calculated using method C of the *CytoSpec* software. With method C the area under the curve is integrated in the range between two defined frequency values (P1 and P2) using a trapezoidal baseline correction. The ratio of amide I to amide II band was calculated using method C/G. The method C/G enables the calculation of the ratio of two peak intensities, each of which has been determined by integration between two defined frequencies (P1 and P2, P3 and P4), as described above. The frequency ranges were chosen so that band overlaps are avoided, especially the overlap between the amide II band with the relatively strong resin peak at about 1512 cm^−1^. Table S3 gives details of the frequency limits chosen for each *CytoSpec* method (see Supplementary Table S3 online).

Further information on the processing and evaluation of Sy-2D-FTIR data with regard to the assessment of parchment degradation are given in^47^ and the Supplementary (see Supplementary Information S.2, S.3 and Supplementary Table S2, S3 online).

## Supplementary Information

Supplementary Information.

## Data Availability

The datasets generated during and/or analysed during the current study are available from the corresponding author on reasonable request.
