# Author Correction: Proton beam irradiation induces invisible modifications under the surface of painted parchment

**DOI:** 10.1038/s41598-022-06628-3

**Published:** 2022-02-08

**Authors:** Katharina Müller, Zita Szikszai, Ákos Csepregi, Róbert Huszánk, Zsófia Kertész, Ina Reiche

**Affiliations:** 1 IPANEMA, Ancient Materials Research Platform, USR 3461 CNRS/MC/UVSQ/MNHN, BP48 Saint‑Aubin, 91192 Gif‑sur‑Yvette, France; 2 Rathgen-Forschungslabor, Staatliche Museen zu Berlin, Stiftung Preußischer Kulturbesitz, Schloßstraße 1a, 14059 Berlin, Germany; 3 Institute for Nuclear Research (ATOMKI), Bem tér 18/c, 4026 Debrecen, Hungary; 4 Ph.D. School in Physics, University of Debrecen, Debrecen, Hungary; 5 PSL University, ENSCP, Institut de Recherche de Chimie Paris – Centre de Recherche et de Restauration des Musées de France, UMR 8247 CNRS/MC, 14 quai François Mitterrand, 75001 Paris, France; 6 New AGLAE, FR 3506 CNRS/MC, C2MRF, 14 quai François Mitterrand, 75001 Paris, France

Correction to: *Scientific Reports* 10.1038/s41598-021-02993-7, published online 07 January 2022

The original version of this Article contained an error in the Acknowledgments section.

“We acknowledge financial support from the European infrastructure projects IPERION CH (654028) and IPERION HS (654028). The samples in this study originate from fresh parchment prepared from calfskin. They were provided by the colleagues Margrit Hundertmark, Julia Bispinck-Rossbacher, Everardus Overgauuw from the collection of the Staatsbibliothek zu Berlin (SBB)—Stiftung Preußischer Kulturbesitz (SPK) in the framework of the Mary of Guelders research program founded by the Ernst von Siemens Kunst Stiftung (Martin Hoernes). The SBB is thanked for having provided permission to include a photograph of the colorful illustrated folio of the Prayer book of Mary of Guelders. Raw mock-up samples were prepared by Ellen Egel, former Rathgen research laboratory (RRL), Staatliche Museen zu Berlin (SMB)-Stiftung Preußischer Kulturbesitz (SPK) now Brandenburgisches Landesamt für Denkmalpflege for the Mary of Guelder project. The RRL staff members, especially Sabrina Buchhorn and Sonja Tesche, are acknowledged for the administrative support of this research program. HZB BESSY II is thanked for providing beamtime at the IRIS beamline in the framework of the proposal no. 17104894-ST/R. Liljana Puskar, Ulrich Schade, the team of the IRIS beamline, as well as Ellen Egel, Cristina Lopes Aibéo (RRL) are kindly acknowledged for their support during beamtime at IRIS beamline. We thank Friederike Fless, president of the DAI, for the interesting discussions on this project.”

now reads:

“We acknowledge financial support from the European infrastructure projects IPERION CH (654028) and IPERION HS (871034). The samples in this study originate from fresh parchment prepared from calfskin. They were provided by the colleagues Margrit Hundertmark, Julia Bispinck-Rossbacher, Everardus Overgauuw from the collection of the Staatsbibliothek zu Berlin (SBB)—Stiftung Preußischer Kulturbesitz (SPK) in the framework of the Mary of Guelders research program founded by the Ernst von Siemens Kunst Stiftung (Martin Hoernes). The SBB is thanked for having provided permission to include a photograph of the colorful illustrated folio of the Prayer book of Mary of Guelders. Raw mock-up samples were prepared by Ellen Egel, former Rathgen research laboratory (RRL), Staatliche Museen zu Berlin (SMB)-Stiftung Preußischer Kulturbesitz (SPK) now Brandenburgisches Landesamt für Denkmalpflege for the Mary of Guelder project. The RRL staff members, especially Sabrina Buchhorn and Sonja Tesche, are acknowledged for the administrative support of this research program. HZB BESSY II is thanked for providing beamtime at the IRIS beamline in the framework of the proposal no. 17104894-ST/R. Liljana Puskar, Ulrich Schade, the team of the IRIS beamline, as well as Ellen Egel, Cristina Lopes Aibéo (RRL) are kindly acknowledged for their support during beamtime at IRIS beamline. We thank Friederike Fless, president of the DAI, for the interesting discussions on this project.”

The original Article has been corrected.

