# Supplementary material for: Proton beam irradiation induces invisible modifications under the surface of painted parchment
Source: Sci Rep. 2022 Jan 7;12:113. doi: 10.1038/s41598-021-02993-7 (PMC8741965; doi:10.1038/s41598-021-02993-7)
Supplement: Supplementary file 1 — Supplementary Information. [file 41598_2021_2993_MOESM1_ESM.pdf]

Supplementary Information

Proton beam irradiation induces invisible modifications under the surface of painted parchment

Katharina Müller[1,2], Zita Szikszai[3], Ákos Csepregi[3,4], Róbert Huszánk[3], Zsófia Kertész[3] and Ina Reiche[2,5,6*]

[1] IPANEMA, Ancient Materials Research Platform, USR 3461 CNRS/MC/UVSQ/MNHN, BP48 Saint-Aubin, 91192 Gif-sur-Yvette, France

[2] Rathgen-Forschungslabor, Staatliche Museen zu Berlin, Stiftung Preußischer Kulturbesitz, Schloßstraße 1a, 14059 Berlin, Germany

[3] Institute for Nuclear Research (ATOMKI), Bem tér 18/c, 4026 Debrecen, Hungary

[4] University of Debrecen, PH.D. School in Physics, Debrecen, Hungary

[5] PSL University, ENSCP, Institut de recherche de Chimie Paris – Centre de recherche et de restauration des musées de France (C2RMF), UMR 8247 CNRS/MC, 14 quai François Mitterrand, 75001 Paris, France

[6] New AGLAE, FR 3506 CNRS/MC, C2MRF, 14 quai François Mitterrand, 75001 Paris, France
[*]Corresponding author

The following details complements the section 'results'

S.1

Structural or chemical degradation of parchment assessed by Sy-2D FTIR mapping

The following shows the results of the Sy-2D FTIR mapping for the non-irradiated parchment reference sample. These data supplement the results obtained for the irradiated parchment sample (W7-A2, beam fluence of 0.5 μC/cm^2) discussed in the main manuscript.

Figure S1 presents a microscopic image of the sample thin section (Fig. 1a), selected FTIR spectra (Fig. S1b) and the obtained Sy-FTIR 2D distribution maps for characteristic vibration bands (Fig. 1c-f). The FTIR spectra shown are each representative of different zones in the thin section in which either only parchment (collagen) or epoxy resin was present or both of them overlapped. Table S1 summarizes the most characteristic peaks found in these spectra and their assignments to specific collagen type I and epoxy resin vibrations.

Tab. S1: Assignments of the characteristic vibration bands observed at various locations on the sample cross-sections of the parchment reference and the irradiated parchment sample (W7-A2) by Sy-FTIR 2D mapping [1-4]. Abbr.: Int. = intensity, v = very, s = strong, m = medium, br = broad, w = weak.

**Sample**	**Frequency**	**Int.**	**Vibration**	**Assignment**	
Parchment reference	1647 cm^{-1} /1655 cm^{-1}	vs	C=O stretching vibrations	amide I	collagen
	1546 cm^{-1}	vs	in plane N-H bending, C-N and C-C stretching vibrations	amide II	collagen
	about 3300 cm^{-1}	m, br	O-H and N-H stretching vibrations	amide A / OH groups	collagen / water
	2976 cm^{-1}, 3080 cm^{-1}	vw	C-H stretching modes of -CH_2 and -CH_3 groups	peptide chains	collagen
	1512 cm^{-1}, 1610 cm^{-1}	s (w)	C-C (and C=C) stretching vibration	aromatic rings	epoxy resin
	1246 cm^{-1}, 1184 cm^{-1}	m, w	C-O stretching vibrations	ethers and/or esters	epoxy resin
	1036 cm^{-1}, 831 cm^{-1}	w	C-O-C stretching vibrations	ethers and aromatic rings	epoxy resin
W7-A2	1647-55 cm^{-1}	vs	C=O stretching vibrations	amide I	collagen
	1547-50 cm^{-1}	vs	in plane N-H bending, C-N and C-C stretching vibrations	amide II	collagen
	3300-30 cm^{-1}	m, br	O-H and N-H stretching vibrations	amide A / OH groups	collagen / water

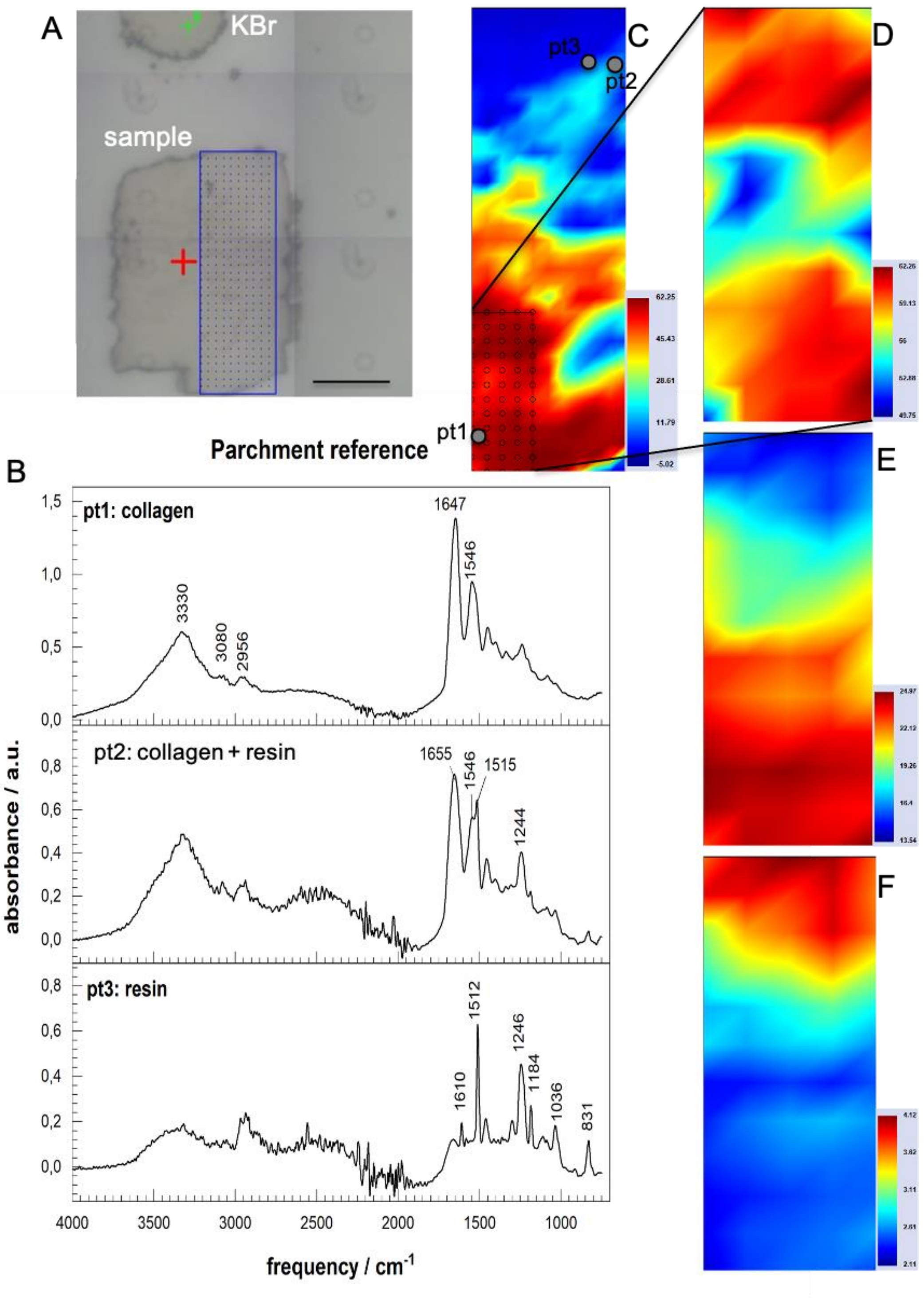

Figure S1: Results of Sy-FTIR 2D mapping on the parchment reference samples. A: Optical micrograph of the thin section placed in a diamond pressure cell with the scanned area indicated (blue rectangle). Scan parameters: 11 x 33 points, aperture 12 x 12 µm^2, step size 10 µm. KBr grains were added to measure the background. B: Selection of characteristic IR spectra (pt1: collagen, pt2: superposition of collagen and epoxy resin, pt3: epoxy resin. For assignment of vibration peaks see Tab. S1. C-F: Sy-FTIR 2D chemical maps representing the intensity distribution of: C) amide I band. The black rectangle indicates the region-of-interest (ROI) used for semi-quantitative evaluation. D) Amide I band within the ROI. E) Amide II band within the ROI. F) Intensity ratio of amide I to amide II bands within the ROI.

Figure S1 also shows the chemical distribution maps of the amide I and amide II band intensities and the ratio of the band intensity of amide I to amide II. The chemical map of the entire sample appears very heterogeneous (Fig. S1c). The intensity of the amide I band is high in the lower left part (red area, point 1) and low in the upper part (blue area, point 2). Instead, epoxy resin is predominantly present in this area (point 3). This indicates heterogeneities or holes in the parchment tissue. For the semi-quantitative evaluation of the Sy-2D-FTIR data, a region of interest (ROI) was defined in which the amide I band intensity appears to be relatively homogeneous. The results of the semi-quantitative evaluation are summarized in Tab. 2 in the main manuscript. Nevertheless, the calculated intensities vary between 5 to 16 % relative standard deviation for the two amide bands. This results in relatively large fluctuations in the intensity ratios between the upper and the lower part of the ROI (Fig. S1f).

The relatively strong resin peak at about 1512 cm^{-1} is overlapping with the amide II band (Fig. S1b). This was taken into account when setting the frequency limits for the semi-quantitative evaluation of the FTIR maps.

The following details complements the section 'materials and methods':

S.2

Evaluation and interpretation of FTIR spectra

Relative positions and intensities of specific vibration bands of parchment found in IR spectra are indicative of the state of preservation of collagen, the main constituent of the parchment. In this study the following vibration bands were considered for this purpose:

a) Amide I vibration band of collagen around 1650 cm^{-1}, which is mainly assigned to C=O stretching vibration (70-85%) and a small proportion of C-N stretching and N-H in plane bending vibrations.

b) Amide II vibration band of collagen around 1550 cm^{-1}, which is assigned to in plane N-H bending (40-60%), C-N stretching (18-40%) and the C-C stretching vibrations (about 10%).

c) Broad vibration band in the water region around 3400 cm^{-1}, which can be assigned to O-H and/or N-H stretching vibrations (water and/or amide A), respectively. A second water vibration band is overlapping with the amide I band at around 1650 cm^{-1}.

d) C=O stretching band in the frequency region between 1700 and 1750 cm^{-1}.

Collagen denaturation to gelatine, hydrolysis and oxidation of the polypeptide chain are the main degradation phenomena of collagen, which can be observed by FTIR analyses. These phenomena can be characterized by specific tendencies in the FTIR spectra of parchment, as described in Tab. S2 [1-2, 5].

Table S2: Characteristic aging phenomena observed for parchment collagen by FTIR spectroscopy [1].

**Characteristic aging phenomena for parchment collagen**	**Observations in the FTIR spectra**
1.) Changes in the collagen structure:	
- Unfolding of the triple helix (denaturation, gelatinization)	Shift of amide II band to lower frequencies: ca. 1550 cm^{-1} to 1530 cm^{-1}, resulting in an increase of $\Delta\nu$ (ν (amide I) – ν (amide II))
2.) Chemical changes:	
- Hydrolysis of polypeptide chain	Increase of OH band intensities at ca. 1650 $cm^{-1}$and 3400 cm^{-1}, resulting in an increase of amide I band intensity due to the overlapping with OH bands, and thus in an increase of the intensity ratio of amide I to amide II bands
- Oxidation of polypeptide chains: formation of carbonyl compound	Appearance of an additional C=O band between 1750 and 1700 cm^{-1} (shoulder at the amide I band)

S.3

Data treatment for Sy-2D-FTIR mapping

The obtained Sy-2D-FTIR mapping data were evaluated using *CytoSpec* software. Prior to calculating band intensities, the spectra were subjected to the following pre-treatment procedure: cutting off the frequency range below 750 cm^{-1}, baseline correction using polynomial fit 2nd order, smoothing (*Savitzky-Golay,* 11 smoothing points). The intensities of the amide I, amide II and amide A (or water) bands were calculated using method C of the *CytoSpec* software. With method C the area under the curve was integrated in the range between two defined frequency values (P1 and P2) using a trapezoidal baseline correction. The ratio of amide I to amide II band was calculated using method C/G. The method C/G enables the calculation of the ratio of two peak intensities, each of which has been determined by integration between two defined frequencies (P1 and P2, P3 and P4), as described above. The frequency ranges were chosen so that band overlaps were avoided, especially the overlap between the amide II band with the relatively strong resin peak at about 1512 cm^{-1}. Table S3 gives details of the frequency limits chosen for each *CytoSpec* method.

Table S3: Definition of frequency limits for the integration of peak intensities of amide vibration bands during 2D FTIR data processing using *CytoSpec* software.

**Vibration bands**	**Choice of frequency limits**			
	**Method C**	**Method G**	**Method C/G**	
**amide I**	P1 = 1596 cm^{-1}<br>P2 = 1730 cm^{-1}	P3 = 1596 cm^{-1}<br>P4 = 1730 cm^{-1}		
**amide II**	P1 = 1490 cm^{-1}<br>P2 = 1596 cm^{-1}	P3 = 1490 cm^{-1}<br>P4 = 1596 cm^{-1}		
**amide I / amide II**			P1 = 1596 cm^{-1}<br>P2 = 1730 cm^{-1}	P3 = 1490 cm^{-1}<br>P4 = 1596 cm^{-1}
**water<br>(and/or amide A)**	P1 = 3180 cm^{-1}<br>P2 = 3530 cm^{-1}			
**water / amide I**			P1 = 3180 cm^{-1}<br>P2 = 3530 cm^{-1}	P3 = 1596 cm^{-1}<br>P4 = 1730 cm^{-1}

References

1. Derrick, M. Evaluation of the State of degradation of Dead Sea Scroll samples using FT-IR spectroscopy. *The book and Paper Group Annual* **10,** 49-65 (1991).

2. Badea, E. et al. Study of deterioration of historical parchments by various thermal analysis techniques complemented by SEM, FTIR, UV-VIS-NIR and unilateral NMR investigations. *J. of Thermal Anal. And Calorimetry* **91,** 17-27 (2008).

3. Manfredi, M., Bearman, G., France, F., Shor, P., Marengo, E. Quantitative multiscale imaging for the detection of parchment aging caused by light: A comparison with ATR-FTIR, GC-MS and TGA analyses. *Int. J. of Conservation Science* **6,** 3-14 (2015).

4. Nicolic, G. et al. Fast Fourier Transform IR Characterization of Epoxy GY Systems Crosslinked with Aliphatic and Cycloaliphatic EH Polyamine Adducts. *Sensors* **10,** 684-696 (2010).

5. Kennedy, C.J. and Wess, T.J. The structure of collagen within parchment – a review. *Restaurator* **24,** 61-80 (2003).
